# Data-driven strategies in operation management: mining user-generated content in Twitter

**DOI:** 10.1007/s10479-022-04776-3

**Published:** 2022-06-10

**Authors:** Jose Ramon Saura, Domingo Ribeiro-Soriano, Daniel Palacios-Marqués

**Affiliations:** 1 Rey Juan Carlos University, Madrid, Spain; 2 Universitat de Valencia, Valencia, Spain; 3 Universitat Politècnica de València, Valencia, Spain

**Keywords:** Data-driven strategies, Operation Management, User-generated content, Twitter

## Abstract

In recent years, the business ecosystem has focused on understanding new ways of automating, collecting, and analyzing data in order to improve products and business models. These actions allow operations management to improve prediction, value creation, optimization, and automatization. In this study, we develop a novel methodology based on data-mining techniques and apply it to identify insights regarding the characteristics of new business models in operations management. The data analyzed in the present study are user-generated content from Twitter. The results are validated using the methods based on Computer-Aided Text Analysis. Specifically, a sentimental analysis with TextBlob on which experiments are performed using vector classifier, multinomial naïve Bayes, logistic regression, and random forest classifier is used. Then, a Latent Dirichlet Allocation is applied to separate the sample into topics based on sentiments to calculate keyness and *p*-value. Finally, these results are analyzed with a textual analysis developed in Python. Based on the results, we identify 8 topics, of which 5 are positive (Automation, Data, Forecasting, Mobile accessibility and Employee experiences), 1 topic is negative (Intelligence Security), and 2 topics are neutral (Operational CRM, Digital teams). The paper concludes with a discussion of the main characteristics of the business models in the OM sector that use DDI. In addition, we formulate 26 research questions to be explored in future studies.

## Introduction

In the last decade, the development of new technologies has boosted business models and marketing strategies globally (Lee & Carter, 2011). Accordingly, the business ecosystem has sought to better understand new ways of automating, collecting, and analyzing data. These actions are developed to make predictions and offer added value to corresponding markets (Qiu et al., 2013). In this paradigm, technologies, artificial intelligence, blockchain, cloud computing, and Internet of Things (IoT) enable companies to develop business models focused on what is known as data-driven innovation (DDI) (Trabucchi & Buganza, 2019; Adamides & Karacapilidis, 2020).

DDI has become widely used in many organizations where decisions are made based on Big Data processes (de Camargo et al., 2018; Hossain et al., 2020). The main objectives of DDI include offering better products and services, improving communication actions, and optimizing the cost of investments (Jetzek et al., 2014). Of note, business changes boosted by the collection and management of data are performed not only on the performance level (Akter & Wamba, 2016), but also on the organizational level (Saura et al., 2021).

As argued by Sorescu (2017), data innovation has been among major emerging drivers to promote new products and services and to generate new business opportunities based on the digital ecosystem. These changes on both performance and organizational levels (Grant, 1996) have led to an increase in the relevance of data innovation in operations management (OM) (Duan and Edwards, 2018; Lins et al., 2021) as a new form of business development to improve results. However, none of the previous studies has identified the main strategies and characteristics of DDI applied in OM business models. Yet, these new strategies are destined to transform the OM industry in the next decade (Thomé et al., 2016; Awan et al., 2021).

Furthermore, like other business areas, OM (Barney, 1991) has been significantly influenced by data-centric decision-making (Akter et al., 2019) and the adoption of strategies in a data-based ecosystem (Yang et al., 2007). OM focuses on the creation of goods and services (De Menezes et al., 2010). Accordingly, OM encompasses the development of plans, organization, coordination, and control of information sources and flows to produce a company’s products and services (Toktay et al., 2000) from data innovation (Tiberius et al., 2021). Therefore, OM has a direct impact on companies’ products and services (Helfat & Peteraf, 2009).

As noted by Pryke (2009) and Farahani & Rezapour (2011), OM is influenced by what is referred to as ‘four Vs’: volume, variety, variation, and visibility. As concerns volume, OM organizes and coordinates large volumes of productions, business structures, as well as the processes that improve decision-making for managing the volume of orders of products, services, and their production (Ransbotham & Kiron, 2017; Liu et al., 2020).

Importantly, using DDI, the analysis and collection of data that a company works with to generate a large volume of orders and production can be automated (Raisch & Krakowski, 2021). Likewise, DDI can be used to predict the volume of goods and services procurement (Johnson et al., 2021). In terms of variety, the actions that a company can perform vary according to each industry (Lakshmi & Bahli, 2020).

However, using DDI, companies can optimize their non-profitable actions, identify new opportunities concerning the services they offer, and measure the efficiency of these services for organizations’ performance (Davenport, 2013; Saura, 2020). Of note, according to the variation in OM, the variety of actions performed by a company based on both economic and production possibilities can be optimized with data-centric strategies (Yue et al., 2014).

In addition, applying data-driven algorithms makes it possible to identify price variations or market strategies (Breidbach & Maglio, 2020). Regarding visibility, experience in OM is usually managed based on operation processes that provide visibility to the actions performed by companies and that allow users to have contact with those companies. In this respect, Hartmann et al., (2014) reported that the use of data can improve visibility of companies and optimize both customer experience and customer journey. Furthermore, as highlighted by Araz et al., (2020), OM and the use of data-centric strategies are relevant for short-term process optimization.

As argued by Thomé et al., (2016) and Awan et al., (2021), the influence of data-centric technologies and strategies in OM should be thoroughly investigated. Awan et al., (2021) proposed to explore the main strategies that should be developed in OM using data-driven strategies, data-based business models, or data-based tools, as well as to measure the influence of technologies such as Big Data (Wamba et al., 2017) and Artificial Intelligence in OM to improve the performance of business processes. Focusing on these contributions, in this study, we bridge this gap in the literature by identifying the main data technologies of new business models based on data in OM and proposing correct uses for their development in different industries (Saura et al., 2021a).

While the main challenges and objectives of data innovation theories highlight the influence of innovation on organization culture, technological sophistication, and commercialization of new products, among others (Saura, 2021; Goel, 2022), the present study aims to understand the impact of data innovation on the development of new business models for OM by identifying its characteristics according to the sentiment of user-generated data (UGD) in Twitter. Therefore, the originality of the present investigation lies in the scarcity of previous research on the characteristics of new DDI business models in OM using Twitter as a source of data and in applying text-mining techniques.

To this end, in the present study, we also develop an original method based on DDI approaches, which reinforces the originality of the present study. More specifically, we first develop a novel method that uses data-mining techniques to identify insights about the characteristics of new busines models in OM based on Twitter-based UGD. Accordingly, methods based on Computer-Aided Text Analysis (CATA) are used to validate results. Specifically, we employ sentimental analysis with TextBlob on which experiments are performed using vector classifier, multinomial naïve Bayes (MNB), logistic regression (LG), and random forest classifier (RFC). Then, a Latent Dirichlet Allocation (LDA), a topic modeling algorithm, is applied to separate the sample in topics based on the sentiment and to calculate keyness and *p* values. Finally, these results are analyzed with a textual analysis developed in Python.

To reach the objectives and address the problems applying a novel method in OM research field, this study addresses the following two research questions: RQ1: *What are the characteristics of business models in OM focused on DDI strategies*? RQ2: *What are the main sentiments in UGC about the characteristics of business models in OM that use DDI*?

The remainder of this paper is structured as follows. Section 2 presents a literature review of relevant past research on OM. Section 3 describes the methodology used in the present study. The results are reported in Sect. 4. Finally, in Sect. 5, the results are discussed, while Sect. 6 draws main conclusions and outlines implications of our findings.

## Literature review

UGD as a data source based on OM has been widely used in the literature. As indicated by Saura et al., (2021a), UGD consists of both user-generated content (UGC) and user-generated data (UGD). In addition, UGD is a valid source of data to extract insights and thereby assist organizations in their decision-making or optimizing their actions on the Internet. For instance, Yuan et al., (2018) used an algorithm to make predictions on eCommerce sales performance using topic modeling and sentiment analysis. Likewise, in a study that explored investments from the OM perspective and the influence of Big Data, Liu & Yi (2018) showed how the use of company information processed with Big Data analytics can help to optimize investment processes and ratios.

In another relevant study, Chae (2015) used the hashtag #supplychain to extract the main practices that can be applied in research, thus using UGC as a source. Similarly, Liu et al., (2019) analyzed the challenges and risk factors for mass customization in OM using as data source platforms and social networks (Twitter among them as one of the main sources of information). Based on the results, the authors argued for using new technologies for OM in fashion systems.

Furthermore, in an analysis of Twitter messages, Gruber et al., (2015) evaluated the states of crisis in OM, as well as the leadership of executives and members of the company’s top management and the actions they take on social networks. The results of this study revealed the relevance of studying Twitter-based UGC in Twitter from the management perspective on the company level.

Likewise, in a case study based on OM, Liao et al., (2021) highlighted the influence of opinion leaders in social networks and innovation in communities. The authors emphasized that Twitter is a valid tool to conduct sentiment analysis and explored its influence on the management of communities on the Internet (Bruns et al., 2014).

In another study on exposure to the impact of opinions on social networks, Rad et al., (2018) analyzed UGC linked to opinions about news. The authors explored how these opinions influence the product lifecycle and linked them to companies’ OM strategies. According to the results of this study, modeling and simulation can help to establish future mechanisms to improve both data-driven and operational strategies of a company.

Finally, Giannakis et al., (2020) used social networks to find out patterns for the development of new business products using consumer sentiment analysis. The authors highlighted the importance of using Big Data analytics (Akter et al., 2016) and artificial intelligence (AI) (Waller & Fawcett, 2013) to improve operation efficiency. A summary of previous studies briefly reviewed above is provided in Table 1.

**Table 1:** Previous studies

Authors	Description
Liao et al., (2021)	This study explored the influence of opinion leaders on social networks (e.g., Twitter) to understand how communities can influence OM by performing sentiment analysis on UGC.
Liu et al., (2019)	This study investigated the main factors to establish challenges for mass customization in OM. The authors used social platforms and networks, including Twitter, as the sources of information.
Giannakis et al., (2020)	This study used UGC on social networks to identify patterns to develop new business products using consumer sentiment analysis.
Chae (2015)	In this study, the hashtag #supplychain was used to extract insights about this industry and to improve processes of management and organization.
Chan et al., (2017)	This study measured and evaluated the role of social media data in operations and production management to improve strategic decision-making from the theoretical and practical perspectives.

Source: The authors

As can be seen in Table 1, there have been diverse previous studies on social networks that sought to identify new forms and characteristics of OM. However, although the UGC has been used as a valid data source in these studies, no academic contribution to date has aimed to identify the characteristics of OM in relation to the main data-driven strategies used by companies. Therefore, by identifying the main characteristics of OM’s business models and linking them to data-centric strategies, this study covers a gap in the literature that has overlooked using a data mining technique to analyze the data from Twitter and theorizing the results for the OM industry.

## Methodology

In recent years, data-mining techniques have come to be extensively used to extract insights and generate knowledge (Verbeke et al., 2012). Following this trend, the present study uses Twitter-based UGD to create knowledge on OM (Franco & Esteves, 2020). To validate this approach, we focus on the use of computer-aided text analysis tools under the CATA framework (Short et al., 2010). According to CATA, validation can be built using computer-aided text analysis tools applied to different industries; then the extracted insights can be used to define major theoretical constructs of interest.

Following Pollach (2012) and McKenny et al., (2018), and aiming to build theory from the data extracted using data-mining approaches, we used the following three methodologies: (1) sentiment analysis with TextBlob (Vijayarani & Janani, 2016), on which experiments with SPC, MB, LG and RFC (Hiremath & Patil, 2020) were validated; (2) LDA, which was used to separate the sample into topics of different emotional valence (Thorsrud, 2020); and (3) textual analysis approach (Krippendorff, 2018) will be applied. The use of this methodology applied to OM opens up a new line of research focused on data. The originality of our methodology will allow other researchers to apply it to identify insights and create knowledge in relation to different areas of OM and combine knowledge discovery, data mining and information sciences in the OM research field. In what follows, we provide further detail on these approaches.

### Data sampling

In order to collect information related to OM and innovation in product development and business models, we collected the following two types of data: (1) tweets with the hashtags #OperationManagement and #Innovation; this was done to obtain information of innovations in OM that were subsequently linked to product development or innovation business models; and (2) tweets with the hashtags #OperationManagement and #Data; these data were collected to obtain insights in relation to product development and innovation in business models based on the use of data and its linkage to DDI (Bermingham and Smeaton, 2011; Kontopoulos et al., 2013; Kim et al., 2018).

Tweets were collected from Twitter API during 3 months, as the volume of UGC does not exceed the standard measure of 5.000 tweets per day (Mittal et al., 2021). The data collection process started on April 1, 2021, and ended on June 15, 2021. The total sample amounted to n = 43.427 tweets. After the sample filtering and debugging process, Python and Pandas libraries were used to exclude from the dataset the tweets that did not exceed 50 characters, had URLs and symbols, as well as duplicate tweets or RTs. After filtering, a total of *n =* 32.283 valid tweets were included in the final dataset.

### Sentiment analysis with TextBlob

Sentiment analysis is a methodological approach that can classify a sample of 32.283 tweets into subsamples containing data expressing different sentiments. These data can be composed of pieces of text, documents, or other types of input (Xia et al., 2015). Sentiment analysis can be conducted using different programming languages (van Atteveldt et al., 2021).

Upon sentiment analysis, the data are divided into categories that express different emotions (Hussein, 2018). In the present study, we focused on the text; therefore, images, emoticons, videos, and other multimedia elements were discarded from the tweets. This was done because the methodological approaches were used to analyze text, rather than multimedia elements (Medhat et al., 2014; Kumar et al., 2015).

An effective tool that has been effectively used for the development of sentiment analysis in the past and that was used in the present study is TextBlob, which was built in NLTK and Patterns (Hardeniya et al., 2016). Of note, while sentiment analysis can identify positive, negative, or neutral sentiments, among its limitations and challenges when categorizing the analyzed content are connotations and irony, as algorithms can barely recognize and classify these characteristics of the language (Gao et al., 2019).

Accordingly, in order to increase the quality of our results, we used the Texblob algorithm taking into account the considerations articulated by Bermingham and Smeaton (2011). Following the indications proposed by Bermingham and Smeaton (2011), polarity was classified from − 1 to 1, while the subjectivity values ranged from 0 to 1. The algorithm was trained a total of 587 times using tweets that manually classified by researchers in the form of text inputs. This was followed by the classification of the tweets into those expressing positive, negative, and neutral sentiments. According to Cherif et al., (2016), the more trained a machine-learning algorithm is, the greater are its predictive capacity and accuracy.

To determine the number of times to train the algorithm, we followed the indications formulated in Li & Zhou (2007). Once the algorithm was trained, four validations that focused on experiments with different development technologies for sentiment analysis in TextBlob were performed. Specifically, the database was experimented and validated with the following calcification models: SVC, MNB, LR y RFC. The results were validated with the analysis of the indicators precision, recall, f1-score, and support, which were also considered in terms of macro average and weighted average.

### Topic modeling

LDA is an algorithm that makes it possible to identify topics on a sample (Thorsrud, 2020). This algorithm is a probabilistic assumption that can be developed using Gibbs sampling in the Mac version. LDA enables identification of different topics in a sample based on the statistical understanding of the words’ location and repetition in a dataset. LDA classifies documents (Hagen, 2018) into topics according to their relevance. The data in this case can be blocks of text in the form of reviews, Tweets, social media posts, and so forth (Park & Oh, 2017).

Before applying sentiment analysis to the Twitter database, the topic modeling algorithm was applied to three different databases. In the first one, LDA was applied to obtain positive topics, and then the process was repeated with negative and neutral tweets. According to Chen (2017), the LDA focuses on understanding of frequency and positioning of the words within this database; the output of this analysis is the automatic grouping of words in the database. Upon topic identification, all topics in a database are labelled using 10–20 most frequent words in each specific topic. While LDA has been widely used to analyze different datasets, none of the previous studies has used this algorithm to explore data about OM and DDI.

### Textual analysis

Textual analysis, which evolved from content analysis proposed by Burrows (2014), is based on the assumption that the weight and word opposition can determine the meaning of patterns not manually identified by users (Krippendorff, 2018). Textual analysis measures the relevance of keywords found in the text to understand what insights and patterns can be identified from the analyzed topics and their sentiments (Chan & Chong, 2017).

In textual analysis, an important value is the weighted percentage (WP), a variable that measures the weight of a keyword in relation to the total database. Furthermore, in content and textual analysis techniques based on NLP, additional techniques can also be used to statistically represent the results of the word that compose a database (Saura et al., 2021). One such techniques is the exploration based on n-grams, i.e. numerical factors that analyze the word prefixes to understand their meaning. Considering that the database is divided into sentiments, the study of n-grams can provide additional insights into the research results. Furthermore, as highlighted by Sidorov et al., (2014) the results can be linked to the values of mutual information, represented by keyness and *p*-value variables. This measure makes it possible understand the occurrence probability of an indicator, correlations among variables, and the weight in terms of the relevance of the analyzed indicators.

## Results

### TextBlob sentiment analysis results

In the present study, the following four experiments were designed to develop the sentiment analysis with TextBlob: SPC, MNB; LG and RFC. Of note, accuracy is a measure that indicates the success of the sentiment modeling. Indeed, accuracy has been widely used in previous studies that used machine-learning as a driver of the experiment (Bermingham and Smeaton, 2011). Here, the higher is the accuracy of the results, the higher is the confidence in the outcome of the research. In the present study, the highest accuracy was found in relation to a Linear SVC Sl. No. 7 (0.869218) and 8 (0.860905). As concerns random forest classifier, the highest accuracy was 0.555445. The accuracy of multinomial Naïve Bayes amounted to 0.737801, while that of logistic regression was 0.837057. Table 2 summarizes the results of the comparison of the results and the different experiments that were conducted.

**Table 2:** TextBlob analysis by experiment

Sl. No.	Model Name	Fold_idx	Accuracy - TextBlob	
0	RandomForestClassifier	0	0.525468	
1	RandomForestClassifier	1	0.537142	
2	RandomForestClassifier	2	0.541182	
3	RandomForestClassifier	3	0.547633	
4	RandomForestClassifier	**4**	**0.555445**	
5	LinearSVC	0	0.864773	
6	LinearSVC	1	0.852851	
7	LinearSVC	**2**	**0.869218**	
8	LinearSVC	3	0.860905	
9	LinearSVC	4	0.861341	
10	Multinomial Naïve Bayes	0	0.713652	
11	Multinomial Naïve Bayes	1	0.706410	
12	Multinomial Naïve Bayes	**2**	**0.737801**	
13	Multinomial Naïve Bayes	3	0.737534	
14	Multinomial Naïve Bayes	4	0.732655	
15	LogisticRegression	0	0.836789	
16	LogisticRegression	1	0.822921	
17	LogisticRegression	2	0.832132	
18	LogisticRegression	**3**	**0.837057**	
19	LogisticRegression	4	0.834027	

Furthermore, different scores of TextBlob were obtained for each of the used models. Table 3 summarizes brief scores in relation to the model used. The comparison between the results of the experiment when working with machine learning is standard in procedures working with sentiment analysis (Li & Liu, 2014). As shown in Table 3, the highest values to the set of accuracy in the results were those corresponding to linear SVC and logistic regression (0.869218 and 0.837057, respectively).

**Table 3:** Brief scores of TextBlob analysis

Sl. No.	Model Name	Scores of TextBlob analysis
1	LinearSVC	0.869218
2	LogisticRegression	0.837057
3	MultinominalNB	0.737801
4	RandomForestClassifier	0.555445

Furthermore, Table 4 shows the classification report for each sentiment: positive, negative, and neutral. The table presents the values of accuracy, recall, f1-score and support obtained for each sentiment. As indicated by Li & Liu (2014), accuracy is a variable that represents the quality of a machine learning model to perform the requested tasks. Likewise, following Supriya et al. (2016), recall reflects the number of parameters that the machine learning model can identify in the database from the total number of inputs. Trofimovich (2016) also highlighted the importance of the f1-score indicator, which is typically used to combine precision and recall into a single value.

This is a practical approach that facilitates a comparison of two performance-centered metrics of accuracy and completeness between various solutions. Furthermore, the support metric shows the predictive ability of the model. As indicated by Bermingham and Smeaton (2011), the macro average measures the total average of the model based on the analyzed variables. Finally, weighted average also measures relativity in terms of weight.

**Table 4:** Classification report of machine-learning model results

Sl. No.	Parameters	Vader			
		precision	recall	f1-score	support
1	Negative	0.75	**0.80**	0.73	20.501
2	Positive	0.83	0.76	0.80	20.201
3	Neutral	0.87	**0.92**	0.91	20.463
4	Accuracy	-	-	0.83	43.641
5	Macro avg	0.81	0.74	0.75	43.641
6	Weighted avg	0.78	0.81	0.83	43.641

As can be seen in Table 4, the highest accuracy indicator obtained for recall was 0.80 for negative sentiments and 0.92 for neutral sentiments.

### Topic-modeling results

Upon application of LDA, a total of 8 valid topics related to the development of new products and business models in OM with the use of DDI were identified in the dataset. As discussed previously, and following Hong and Davison (2010), the 20 most frequent words in each topic were used to create labels for each of the identified topics.

Then, the connections of specific words to sentiments and specific topics were measured. Of note, since LDA is an automated process, researchers have to adapt their criteria in an exploratory and manual way. Likewise, the insights were previously linked to the sentiment analysis process, since LDA was applied to the set of tweets divided according to the sentiments expressed in them. In this way, the topics were identified as positive, negative, or neutral.

Thus, of the total number of modeled topics, Table 5 presents the topics that are relevant to the research questions addressed in the present study. Furthermore, following Ambrosino et al., (2018), we evaluated the relevance of each topic using keyness values. Keyness (Gabrielatos and Marchi, 2011) is a metric that measures the relevance of the topics and is defined as the strength of the link between the topics linked to the log-likelihood score values (Rayson and Garside, 2000). Through the use of keyness (Gabrielatos, 2018), the log-likelihood of > 3.8 was statistically significant when *p-*value < 0.05.

**Table 5:** Topic modeling results

R	Topics	Description	Sent.*	Keyness	*p*-value
1	Automation	Studies the initiatives focused on automating processes related to OM and the organization and structure of the systems in the industry	Positive	733.01	0.045
2	Data	Focuses on the approach to data for decision making, automation of processes according to the type of data, and sources of data collection.	Positive	451.73	0.031
3	Forecasting	Displays information related to new initiatives for forecasting and forecasting demand.	Positive	423.08	0.029
4	Mobile Accessibility	Focuses on the initiatives related to the increase in mobile accessibility to OM-related issues at the organizational level.	Positive	400.97	0.027
5	Employee experiences	Analyzes the treatment and priority offered by companies in relation to OM and the experiences perceived by employees	Positive	361.12	0.026
6	Intelligence Security	Focuses on computer systems that adapt the level of security to companies’ priorities. It also includes computer attacks and cybersecurity strategies	Negative	347.84	0.024
7	Operational CRM	Studies the use of Customer Relationship Management (CRM) in OM, as well as the strategies developed in each case.	Neutral	347.75	0.024
8	Digital teams	It refers to concepts related to Digital Operation Management (DOM), which consists of mobilizing teams online to make business-related decisions	Neutral	338.27	0.021

### Textual analysis results

As discussed previously, textual analysis is used to derive insights based on relevance, frequency, and position of words in a dataset. Textual analysis is the third step in the application of data-mining techniques to create knowledge and extract valid insights (Asarta & Méndez-Carbajo, 2020) to build theory and understand the object under study. In the present study, we analyzed the number of times that keywords were repeated in the data, as well as their WP, as which is an indicator of the relevance and weight of a keyword and similar ones grouped within the same node (grouping words) in the total database (Loughran & McDonald, 2016).

In structuring the results, we followed content analysis directions proposed by Ibrahim and Ahmad (2010) and the NLP conceptual framework (Bos and Markert (2005). Pandas GroupBy in Python was used (McKinney, 2012). Table 6 presents the main words identified as a result of textual analysis, similar words that compose the same node, total frequency of these words, and their WP.

**Table 6:** Grouped keywords by topic

R	Word	Similar words	Frq.	WP
1	Automation	Value of automation, benefits of automation, automation tools, automation processes, among other terms.	7272	15.12
2	Data	Data use, Big Data tools, data operations, data structure examples, data platforms, among other terms.	4286	13.38
3	Forecasting	Forecasting methods, forecasting in management, forecast, forecasting production, among other terms.	3710	11.08
4	Mobile accessibility	Mobile operations, UX operations, mobile management, mobile operations managements, among other terms.	2890	7.71
5	Employee experiences	Clients’ experiences, customer journey, employees’ cases, employees’ performance, among other terms.	2749	7.01
6	Intelligence security	Security operations, incident management, intelligence management, security operations, among other terms.	2035	5.19
7	Operational CRM	Management software, processing operations, self-service management, among other terms.	1987	5.11
8	Digital teams	Members, employees, team meetings, Skype, Zoom, Google Meet, among other terms.	1980	4.98

Finally, in order to understand the structure of the content, and following Sapkota et al., (2015), we used n-grams to obtain additional insights. This approach is common in linguistic studies. An n-gram model can predict the occurrence of a word with the analysis of n-1 previous words. Accordingly, a bigram model (n = 2) works predicts the occurrence of a word given only its previous word (as n – 1 = 1). Table 7 shows the unigrams and bigrams indicators. Of note, as indicated by Daneshvar & Inkpen (2018), the study of n-grams allows enables researchers to analyze strong and stable relationships between different terms. Table 7 presents the main topics identified in the methodological process along with their Rank (R), Frequency (F) of words on the left (FreqL) and on the right (FreqR). Finally, the main collocates are also presented.

**Table 7:** N-grams for the identified collocates

R	Collates for Automation				R	Collocates for Data				
	Freq	Freq L	Freq R	Collocate		Freq		Freq L	Freq R	Collocate
1	7272	3581	1691	Automation	1	4286		2139	2147	Data
2	1491	731	760	Tools	2	2604		1290	1314	Strategies
3	680	357	323	Benefits	3	420		200	220	Decision
4	591	289	302	Process	4	381		186	195	Tools
5	321	149	172	Strategies	5	204		89	115	Platforms
R	Collates for Forecasting				R	Collocates for Mobile Accessibility				
	Freq	Freq L	Freq R	Collocate		Freq		Freq L	Freq R	Collocate
1	3710	1840	1870	Forecasting	1	2890		1399	1491	MobileAcc.
2	2901	1390	1511	Actions	2	891		587	304	Remote
3	1002	510	492	Management	3	705		326	379	Online
4	574	276	298	Production	4	579		390	189	Connected
5	201	99	102	Dashboards	5	102		105	92	Management
R	Collates for Employee experiences				R	Collocates for Intelligence Security				
	Freq	Freq L	Freq R	Collocate		Freq	Freq L		Freq R	Collocate
1	2749	1256	1493	EmployeeExpe.	1	2035	967		1068	IntelligenceSec.
2	766	356	410	Reviews	2	579	267		312	Management
3	508	247	261	Feedback	3	320	149		171	Cyberattacks
4	487	239	248	Performance	4	309	152		157	Investments
5	221	196	25	Examples	5	281	138		143	Privacy
R	Collates for Employee Operational CRM				R	Collocates for Digital Teams				
	Freq	Freq L	Freq R	Collocate		Freq	Freq L		Freq R	Collocate
1	1987	974	1013	OPCRM	1	1980	891		1089	DigitalTeams
2	521	253	268	Systems	2	601	347		254	Makingdecision
3	554	268	286	Tools	3	234	106		128	Meetings
4	251	117	134	Software	4	163	138		31	Shared
5	230	128	102	Organize	5	113	96		17	Tools

## Discussion

In recent years, DDI strategies have been extensively used in the OM sector. Accordingly, organization, structure, and design of DDI strategies have opened up new opportunities for business development and the approach to data-centric decision-making (Wang et al., 2018). However, as argued by Zhang et al., (2019), the influence of DDI on OM and short-term trends remains poorly understood. To bridge this gap in previous research, in the present study, we used data-mining techniques to analyze Twitter-based UGC to identify the main themes relevant for the new business models focused on OM.

Zhao et al., (2016), we identified a total of 8 topics related to OM. The most relevant topic was the positive topic “automation” (keyness = 733.01; *p =* 0.045). With the TA frequency of 7272 and WP of 15.12, this topic reflects the importance that process automation for the OM sector and for the improvement of the relationship with the suppliers or customers. In this respect, our results are consistent with those reported by Tsai et al., (2013).

Another positive topic “Data” (keyness = 451.73; *p =* 0.031), with the TA frequency of 4286 and WP of 13.38, highlights the importance of DDI in the OM sector in terms of actions and strategies that use data for the development and management processes. Similarly, Nwokeji et al. (2015) underscored the importance of data-centric approaches to improve decision-making in OM companies. Furthermore, there is also evidence that data collection and analysis may shape the future of the OM sector (Awan et al., 2021).

Next, the positive topic “forecasting” (keyness = 423.08; *p =* 0.029), with the TA frequency of 3710 and WP of 11.08, shows the relevance of prediction techniques and tools that can be based on artificial intelligence, as well as tactics such as machine learning or Big Data analytics. In line with our findings, Kumar et al., (2021) argued that forecasting and prediction techniques in the OM sector will help to anticipate the challenges and issues that may arise in the OM sector.

Another positive topic identified in our data analysis is “Mobile Accessibility” (keyness = 400.97; *p =* 0.027). With the TA frequency of 2890 and WP of 7.71, this topic highlights the mobility of the sector towards transformation to the mobile ecosystem. Recently, both the organization of tasks and the relationship and communication with customers and suppliers have moved to a mobile-friendly sphere. Likewise, Fragapane et al., (2020) showed that mobile accessibility in OM is essential to make decisions in short time that can determine the added value of one company over another (Yamin & Alharthi, 2020).

The positive topic “Employee experiences” (keyness = 361.12; *p =* 0.026), with the TA frequency of 2749 and a WP of 7.01, indicates that the opinions and experiences of the company’s employees should be prioritized. In line with this finding, Reid & Sanders (2019) argued on the need to promote internal communication with customers in OM so that to ensure a successful relationship between suppliers and services. In an automation and data-centric ecosystem, employees play a key role in company’s relationship with its stakeholders (Pagell & Gobeli, 2009.

In contrast to the positive topics discussed above, the negative topic “Intelligence security” (keyness = 347.84; *p =* 0.024), with the TA frequency of 2035 and WP of 5.19, highlights the challenges of the use of DDI and the collection, analysis and prediction of data in terms of security and privacy of processes in OM. Intelligence security reflects concerns about corporate data leaks, industry cyber-attacks, patents, and trade secret thefts, and so forth (see also Choi et al., 2020). In an increasingly automated and data-centric ecosystem, security and cybercriminal attacks are becoming more common. Therefore, companies should not only adapt their actions to sales forecasts to increase profitability, but also consider the issues that can negatively impact the automation processes, data collection, and data-centric decisions (Saura et al., 2021).

Furthermore, the neutral topic “Operational CRM” (keyness = 347.75; *p =* 0.024), with the TA frequency of 1987 and WP of 5.11, confirms the importance of appropriate management of data processing and online management tools. Operational CRM can organize and structure strategies and, when combined with tools such as analytical CRMs (Schniederjans et al., 2012), the use of artificial intelligence can be a key point in business development (Liu et al., 2018).

Finally, the neutral topic “Digital teams” keyness = 338.27; *p =* 0.021), with the TA frequency of 1980 and a WP of 4.98, reveals the importance of employees’ management and their availability to telework. Recently, COVID-19 pandemic has dramatically transformed a multitude of processes into the digital ecosystem. Therefore, according to Grover et al., (2020), the OM industry must focus on both adapting traditional processes to the digital ecosystem and training its employees to make better decisions and manage processes by teleworking using tools that work directly in the cloud.

Taken together, the results of the present study highlight that the process automation, data-centric strategies, and digitalization of traditional processes to the digital environment will shape the characteristics of the OM sector in terms of integration of DDI strategies (Metallo et al., 2021). Thus, in order to present these contributions applied to the OM industry, in Table 8, we formulate future research questions regarding the use of DDI strategies in OM in relation to the topics identified in this study.

**Table 8:** Future research questions in relation to DDI in OM research

Topic	Research questions	Relative authors
Automation	♣ What are the main automation tools that increase effectiveness in OM? ♣ How does the automation of processes in OM affect the relationship with suppliers or customers? ♣ What are the pros and cons of automating processes and business models in OM with the use of AI or Big Data?	Tsai et al., (2013) Zhao et al., (2016)
Data	♣ What are the main sources of information in OM in relation to the collection and treatment of business process data? ♣ How does the use of data-centric technologies and strategies affect decision-making processes in OM? ♣ Should new OM business models be data-centric to become more profitable?	Awan et al., (2021) Nwokeji et al. (2015)
Forecasting	♣ What are the main prediction techniques used in OM to improve the effectiveness of new business models? ♣ What variables in OM should be studied with prediction techniques? ♣ How can the use of demand prediction algorithms in OM influence the production of products and services?	Kumar et al., (2021) Araz et al., (2020)
Mobile Accessibility	♣ What are the main influences of the development of mobile technology in the manufacturing processes in OM? ♣ What mobile accessibility technologies should the OM industry adopt? ♣ How will communication with customers and suppliers be transformed with the adoption and use of adoption of new mobile accessibility technologies?	Fragapane et al., (2020) Yamin & Alharthi (2020)
Employee experiences	♣ How should OM industry managers propose business models in which employee experiences are perceived as valued? ♣ What are the guidelines for the development of new business models where employees’ experiences and opinions are prioritized? ♣ How should the relationship with stakeholders and communication with employees be linked to improve communication experiences in OM processes?	Reid & Sanders (2019) Pagell & Gobeli (2009)
Intelligence security	♣ How should new forms of data collection be applied in OM? ♣ What DDI-centric business models can be adapted to the OM industry? ♣ What protocols executives should take into account when managing collected data and privacy concerns at a professional and industrial level?	(Saura et al., 2021) Choi et al., (2020)
Operational CRM	♣ How should Operational CRMs be improved to function with AI in OM? ♣ What are the main techniques to apply AI in OM when using Operational CRMs? ♣ What are the key variables and indicators to measure strategies and processes in OM when using Operational CRMs?	Liu et al. (2018) Schniederjans et al., (2012)
Digital teams	♣ Is Industry 4.0 adapted for the adoption of teleworking among its employees in OM? ♣ Do the connectivity of companies and the software and hardware allow incentives to create and communicate between teams? ♣ Do employees have enough tools to work in digital ecosystems and organize teams?	Metallo et al., (2021) Grover et al., (2020)

## Conclusions

In the present study, we applied data-mining techniques (namely, sentiment analysis, LDA and textual analysis) to analyze Twitter-UGC to identify the main characteristics of the new business models focused on DDI in the OM sector. Based on the results, we identified 8 topics: 5 positive (Automation, Data, Forecasting, Mobile accessibility and Employee experiences), 1 negative (Intelligence Security), and 2 topics are neutral (Operational CRM, Digital teams). The results were discussed in terms of the main characteristics of the business models in the OM field that use DDI. We also formulated 26 research questions to be further investigated in the future.

Therefore, our first research question (RQ1: *What are the characteristics of new business models in operation management focused on DDI strategies?)* was answered with the insights of the positive and negative characteristics of the identified topics. In addition, in terms of the relevance of each topic, the automation processes in OM (as the new perspectives in the data-centric business models based on analysis, collection, prediction, and forecasting) reveal the trends in the OM sector when using DDI. Moreover, it is important to point out the influence mobile accessibility in the workplace, as suggested by the importance of both the working teams and employees’ experiences with the use, adoption, and enjoyment of data-centric tools. The new processes in OM focus on, among other functions, connectivity and the use of new technologies to drive risk reduction in production, result measurement, data collection, and demand prediction. The adoption of new strategies of data monitoring and analysis, as well as their optimization, are the main development paths for the new business models in OM.

Furthermore, our second research question (RQ2: *What are the UGC’S main sentiments about the characteristics of new business models in operation management that use DDI?*) was addressed by explaining and discussing the relevance of the identified. Of note, only one topic in our data– intelligence security—was negative. This suggests that cyber-attacks and risks of data leakage should be closely investigated in the future as one of the ways to protect industrial secrets, innovation in business models, and the prediction of future actions in OM. While DDI strategies in the OM sector provide new mechanisms to develop and promote business innovation, if these applications are not appropriately implemented, the risks linked to misuse of artificial intelligence or similar technologies could jeopardize business success in OM.

### Theoretical implications

The present study is a pioneering attempt to apply data-mining analysis tools to analyze Twitter-based UGC in the OM domain. In further research, our results, presented as topics, could be analyzed as new points of knowledge, be tested with empirical methodologies that measure statical significance based on approaches focused on understanding both the adoption of DDI in OM, or be used as constructs or variables in statistical models. In addition, since the present study takes the UGC under CATA as a valid source of data to develop theory, our findings can be extended in future research to improve its accuracy and to identify additional insights in relation to DDI in OM.

Regarding the results, new theoretical investigations should focus on the use of automation actions in OM. Together with data-driven business models and prediction technologies, this focus may cause a massive change in industrial production and transformation policies in the next decade. In addition, the identification of the indicators for employees to be valued in OM must be properly classified and defined. In addition, digital teamwork has become a priority and should be more closely investigated in relation to the development of business models in OM.

Finally, our study can serve as an example for further studies aiming to explore the characteristics of business models from UGC on Twitter. Our results can be replicated using new methods applied to DDI in OM and in other industries.

### Managerial implications

First of all, the results obtained can be used by OM companies as a practical guide to identify the characteristics of new DDI-centric business models. In this way, OM companies can better understand how automation, data, and the mobile ecosystem are driving innovation in OM. Furthermore, based on our findings, practitioners can better understand the relevance of each of the identified topics for the OM field. Furthermore, our results are informative for CEOs and executives in terms of exploring new trends and characteristics of the industry’s adaptation to a data-centric and digital era.

Accordingly, the identified indicators can serve as a guide to improve decision-making in OM when working with DDI. Practitioners should understand the characteristics of new business models in OM and combine practical knowledge with the adoption of technological development. It is equally important to ensure that communication with stakeholders is fluid and constant, since technological development and the adoption of DDI business models drive the development of interactive and connected models. The processes of adaptation to digital change in OM, as well as the use of new platforms for data collection and analysis or production prediction, among others, must attend to the principles, characteristics, and limits of the development capacity of new models.

### Limitations and future research

The limitations of the present study are related to the number of tweets in the sample. Indeed, the more trained are the algorithms, the more reliable will be the results. Therefore, in order to achieve higher accuracy, it is necessary to continuously train the algorithms over time. In addition, despite the use of quantitative analysis approaches, the present study is exploratory, as it takes UGC as a sample. While UGC has been widely used as a valid source of data in many previous studies, the results of the present study should be compared with other data sources, such as opinion surveys or focus groups. Doing so would complement the present results and increase the quality of the identified indicators. In further research, it would be necessary to more closely focus on each of the topics identified in the present study and link them to OM both for the improvement of decision-making and the larger-scale adoption of new DDI-centric business models.
